# Intra-demographic birth risk assessment scheme and infant mortality in Nigeria

**DOI:** 10.1080/16549716.2017.1366135

**Published:** 2017-09-08

**Authors:** Ayo S. Adebowale

**Affiliations:** ^a^ Department of Epidemiology and Medical Statistics, Faculty of Public Health, College of Medicine, University of Ibadan, Ibadan, Nigeria; ^b^ Visiting Academic, Division of Actuary Research, Faculty of Commerce, University of Cape Town, Cape Town, South Africa

**Keywords:** high-risk birth, infant mortality, birth risk assessment scheme, Nigeria

## Abstract

**Background**: Infant mortality (IM) is high in Nigeria. High-risk birth can limit a newborn’s survival chances to the first year of life. The approach used in investigating the relationship between high-risk birth and IM in this study is yet to be documented in Nigeria.

**Objectives**: The Intra-Demographic Birth Risk Assessment Scheme (IDBRAS) was generated and its relationship with IM was examined.

**Methods**: 2013 Nigeria demographic and health survey data were used. Mothers who gave birth in the 5 years before the survey were investigated (*n* = 31,155). IDBRAS was generated from information on maternal age at childbirth, parity and preceding birth interval and was disaggregated into low, medium and high. Data were analysed using the Cox proportional hazard and Brass 1-parameter models (α = 0.05).

**Results**: Infant mortality rate was 88.4, 104.7 and 211.6 per 1000 live births among women with low, medium and high level of IDBRAS respectively. The rate of increase of reported infant deaths between low and high IDBRAS was 0.1932 (*R* ^2^ = 0.5326; p < 0.001). The prevalence of medium- and high-risk birth was 24.6 and 4.2% respectively. The identified predictors of IM were place of residence, marital status and size of the child at birth. The hazard ratio of IM was higher among women with medium (HR = 1.35; 95% CI = 1.22–1.48, *p* < 0.001) and high IDBRAS (HR = 1.73; 95% CI = 1.48–2.02, *p* < 0.001) than among those with low IDBRAS. Controlling for other correlates barely changed this pattern.

**Conclusions**: The risk and level of IM increased as the level of IDBRAS increases in Nigeria. IDBRAS was an important predictor of IM. Maintaining a low level of IDBRAS will facilitate a reduction in IM rate in Nigeria.

## Background

The infant mortality rate has been declining in most developing countries in the past two decades [1]. However, the pace of decline is slow in some countries in sub-Saharan Africa. The ever high childhood mortality in the region is due to numerous reasons, one of which is high-risk birth [2–6]. High-risk birth remains a public health issue of important concern in sub-Saharan Africa because of the salient cultural practices like early marriage and sex preference which promote early childbearing and high birth frequency respectively [7,8]. The mortality and morbidity associated with high-risk birth are enormous and the poor economic conditions in developing countries like Nigeria aggravate the problem. Nigeria is a high-fertility country with a total fertility rate of 5.5 and infant mortality rate of 68 per 1000 live birth [9]. These high demographic parameters classify Nigeria among countries where demographic transition is yet to begin [1]. High-risk birth have been associated with high mortality in many nations [10–12]. In a setting with the best healthcare system and practice, infants’ survival probability is expected to be close to one. However, the interaction between socioeconomic, biological and environmental factors often leads to a decrease in this survival chance [13].

Many factors have been implicated as responsible for a newborn’s risk of dying before age one. Studies have revealed lower utilization of health facilities among pregnant women as an important determinant of infant mortality [14–16]. An infant mortality differential also exists according to place of residence, child’s sex, mother’s level of education and household wealth [17]. Others include number of antenatal visits, presence of a birth attendant, birth size and exclusive breastfeeding [17,18]. Findings from scientific studies have also established a strong relationship between infant mortality and specific demographic factors like maternal age at childbirth, birth order and preceding birth interval [18]. Typically, the early childhood probability of dying is higher for children born to too young or too old mothers, children born after a short birth interval and children born to women who have had more than three births [17,19–21].This is obviously because a very young mother is likely to experience poor pregnancy outcome because of her physical and physiological immaturity. Older women in turn may experience age-related problems such as hypertension, diabetes, cancer, high blood pressure and kidney disease that can influence the infant’s risk of dying [22]. The literature has also shown that inter-pregnancy intervals less than 18 months and longer than 59 months are related to increased risk of poor perinatal outcomes like pre-term birth, low birth weight and small for gestational age birth [20,21]. In a study conducted in Bangladesh for instance, it was established that shorter birth intervals were associated with higher infant mortality after controlling for other important factors [21]. Moreover, a number of studies have revealed that high parity births are more linked to higher infant mortality than low parity births [23–25]. Childhood mortality rates are described as having a U-shaped relationship with birth order, with first-order births and higher-order births experiencing a higher risk of death than middle-order births [23,26].

Each of these factors (maternal age at childbirth, birth order and preceding birth interval) has been established as having an independent influence on infant survival through their effects on maternal health [20–22]. A synergetic effect may also exist between short birth spacing, birth order and young maternal age [17]. This study integrates these three important demographic determinants of infant mortality as a single entity to reveal the impact of the joint effect rather than the individual effects on infant mortality. The entity formed in this context after decomposition is referred to as the intra-demographic birth risk assessment scheme (IDBRAS) and it is the outcome variable of principal focus in this study. With this, mere estimation of the level of IDBRAS during delivery can provide information on the child’s survival risk that the child might be exposed to before the first birthday. This idea has been used for determination of some health indices such as nutritional status using body mass index, hypertension, diabetes using fasting blood sugar and a host of others.

Though several scientific investigations have improved understanding of the causes of childhood mortality in Nigeria [14,15,17], a new approach as incorporated in the current study is clearly necessary. The method discussed here classifies maternal demographic childbirth risks into low or medium or high. A birth risk is one in which some conditions put the mother, the newborn or both at higher than normal risk of dying. Knowledge of IDBRAS may prompt intervention that will enhance the survival chances of the child through close monitoring. This study aims to establish a relationship between IDBRAS and infant mortality in Nigeria. It also identifies the predictors of infant mortality. The adjusted infant mortality rate was also estimated across the IDBRAS categories. The outcome of the study will assist the planners and policy-makers in their decisions to sustain an active framework of infant mortality reduction activities in Nigeria. The developed IDBRAS can also be adapted to other research areas.

## Methods

### Study setting

The study was conducted in Nigeria, Africa’s most populous country. Persistent high levels of fertility (TFR = 5.5), infant mortality (68 per 1000 live births) and maternal mortality (550 per 100,000 women) have been reported in the country in recent times [19,26]. The country has six geo-political zones in which there are 36 states and local government areas. Nigeria is a multi-ethnic country but its major tribes are Hausa/Fulani, Igbo and Yoruba. The main religions of the people are Muslim and Christianity, which are commonly practised in the northern and southern parts of the country respectively. The public healthcare system in Nigeria is managed by the government and is characterized by inadequate health workers, lack of essential drugs and poor equipment supply. In most situations, patients buy their drugs themselves and the harsh economic conditions have prevented people, particularly the poor, from accessing health facilities in Nigeria. In this situation, pregnant women, nursing mothers and children who are most susceptible to diseases, infections and morbidity are most affected.

### Study design and data collection

Nigerian demographic and health survey data were used [27]. In this cross-sectional design population-based study, a multi-stage cluster design approach was used to select women of reproductive age. It was a nationally representative sample. The sampling procedures thus allowed for the data to be analysed to examine health, social and demographic related issues. Complete information about the sampling procedure is available at the measuredhs website for interested readers. The data were recorded based on the birth status of women and thus women who had birth in the last five years were separated from other women in order to examine their infant and childhood mortality experience. This also provides an avenue for the examination of morbidity prevalence among the children and the healthcare-seeking behaviour for them and that of their mothers. In the current study, mothers who had given birth in the 5 years preceding the survey were investigated and such women had to have complete information on the variables that were used in creating the key independent variable. Consequently, the sample size for this study was 31,155.

### Variable description

The dependent variable was infant mortality, which was based on the survival status of children before reaching the end of the first 12 months of life. Thus, if the child was alive at age one, he or she was assigned a code 0, and 1 if dead.

The main independent variable was Intra-Demographic Birth Risk Assessment Scheme (IDBRAS) and this was created from the three demographic variables that mainly put women and children at health risk during pregnancy and childbirth. These are: age of the woman at the birth of the reference child (coded as 10–19, 20–29, 30–39 and 40–49), the child’s birth order (coded as 1, 2–3, 4–6 and 7+) and the preceding birth interval of the child (coded as first birth, <24 months, 24–35 months, 36–47 months and 48+). The coding was based on the pattern used in the demographic health and survey’s report. In each category of these three variables, preliminary percentages of infant deaths were obtained to assign weights to them. The category with the smallest percentage was used to divide the percentage obtained for the other categories by the same variable, *p_i_*_2_ (intra). For instance, regarding birth order, the percentages of infant death were 7.4, 5.8, 6.4 and 9.2% among the 1st, 2–3, 4–6 and 7+ birth order respectively. In this case, the birth order with smallest percentage was 2–3 months. Therefore, 5.8% was used to divide the others to obtain the intra-infant death risk ratio (1.276, 1.000, 1.103 and 1.586). For maternal age at birth of the child, the percentages of infant death were 8.5, 6.3, 6.7 and 8.8% among women aged <20, 20–29, 30–39 and 40–49 years respectively, thus generating intra-infant death risk ratios (1.349, 1.000, 1.063 and 1.397). The percentages of infant deaths were 10.2, 6.3, 4.9 and 5.1% for women who left 0–23, 24–35, 36–47 and 48+ birth intervals respectively. In this case, the intra-infant death risk ratios were 2.082, 1.286, 1.000 and 1.041 respectively. Consequently, the total maximum infant death risk ratio was obtained for all these three main variables as 1.586 + 1.397 + 2.082 = 5.065 and the minimum was 1 + 1 + 1 = 3.000. The mathematical equations relating to the derivation of IDBRAS are as shown in Equations (1–4) below.

(1)
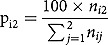

(2)
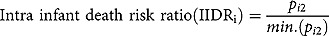

(3)
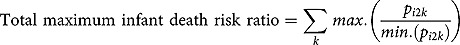

Total minimum infant death risk ratio = 3.000

(4)
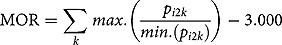

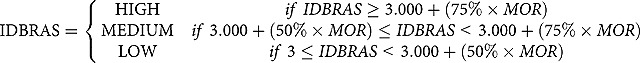

where MOR is the measure of risk. The ‘*i*’ represents the categories in each of the variables used for the computation of IDBRAS and *j* represents the categories of the outcome variable, i.e. infant mortality (No = 1, Yes = 2). Thus *j* = 1 if the response is No and j = 2 if Yes. The cut-off points 50 and 75% were based on second and third quartiles respectively. A woman with IDBRAS of 75% and above is considered as high risk, 50–74.99% as medium and low if otherwise.

There are a number of factors that may potentially confound the relationship between IDBRAS and infant mortality. These are the socio-economic characteristics of the women, which included region of residence, education, place of residence, household wealth, religion, ethnicity and marital status, and environmental characteristics such as cooking fuel, sources of drinking water and toilet facility and health facility access factors at the time of the child’s pregnancy and delivery (tetanus injection during pregnancy, number of ANC visits, place of delivery, prenatal attendant and delivery assistant). To control for possible confounding effects, variables representing these factors were used in multivariate analyses. In order to avoid multicollinearity, a phenomenon where predictor variables are highly correlated, multicollinearity assessment was performed before inclusion of such variables in the regression model.

### Data analyses

Descriptive statistics were used to describe the data across the variables. A Chi-square model and analysis of variance were used to test association between IDBRAS and socio-economic factors. Cox regression was used to examine the relationship between infant mortality and IDBRAS amidst other factors. At this level of analysis, five models were generated. The first model is the unadjusted model, which involved only two variables, infant survival status variable and one independent variable. In the second model, the relationship between infant mortality and IDBRAS was adjusted with the inclusion of socio-economic factors such as region, education, place of residence, household wealth, region, religion and marital status. In the third model, health facility utilization factors were introduced into the equation to examine how their inclusion affected the strength of the relationship between IDBRAS and infant mortality. The child’s related factors were used in the fourth model, while the fifth model is the full model which includes all variables found to be statistically significant in infant mortality in the first model.

The Cox regression procedure is useful for modelling the time to a specified event, based upon the values of given covariates. For each child in the study, time (*t*) starts with a value of zero at birth and is right censored at the first 12 months of life. Meanwhile, a child who is alive and has not reached the age of 12 months at the time of the study is censored, including those whose survival status is unknown. Thus, the cases are those who died between ages zero and 1 year. The indicators of child survival in the analysis are the survival status of the child (alive = 0 or death = 1) and the time (*t*) from age 0 to the timing of death; *t* depends on a characteristics vector, *X* ***_i_***(*X*_1_, *X*_2_,…, *X_n_*). The basic model offered by the Cox regression assumes that the time to event (infant mortality) and the covariates are related through the following equation:

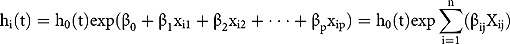

where *h_i_*(*t*) is the hazard rate for the *i* ^th^ case at time *t*; *h*_0_(*t*) is the baseline hazard at time *t*; *p* is the number of covariates; β*_j_* is the value of the *j* ^th^ regression coefficient; *X_ij_* is the value of the *i* ^th^ case of the *j* ^th^ covariate. The hazard function is a measure of the potential for the event mortality to occur at a particular time *t* (any time in or before the first 12 months of life), given that the event is yet to occur. Larger values of the hazard function indicate greater potential for the event to occur. The baseline hazard function measures this potential independently of the covariates. The shape of the hazard function over time is defined by the baseline hazard, for all cases of infant mortality. The covariates determine the overall magnitude of the function. The value of the hazard is equal to the product of the baseline hazard and a covariate effect. While the baseline hazard is dependent upon time, the covariate effect is the same for all time points. Thus, the ratio of the hazards for any two cases at any time period is the ratio of their covariate effects. This is the proportional hazards assumption.

Infant survivorship probabilities were estimated using the Brass 1-parameter logit system. The system used information on the proportion of children dead (*D*(*i*)) to a cohort of women and average parity (*P*(*i*)). A set of multipliers ζ(*i*) = *a*(*i*) + *b*(*i*)*{*P*(1)/*P*(2)} + *c*(*i*)*{*P*(2)/*P*(3)} were used (*a*(*i*), *b*(*i*) and *c*(*i*) are multiplier coefficients selected from West model life tables) to obtain the probability of dying *q*(*x*)* = ζ*(*i*)*D(*i*) and this estimate was adjusted using the equation *Y*(*x*) = α+β*Y*(*s*) given by

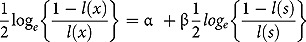

where α and β are constants and β = 1. The logit of the observed is *Y*(*x*) and that of the standard is *Y*(*s*) with their corresponding survivorship probabilities, *l*(*x*) and *l*(*s*). In this study, the Brass African standard was used [28]. The estimated probability of dying was thereafter converted to the infant mortality rate using the equation 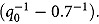


The hazard function *h*(*t*) and survival function *S*(*t*) are mathematically related as;

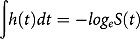

### Ethical clearance

Ethical approval for this study was obtained by the data originators from the Nigeria National Ethics Committee (NHREC/2008/07), functioning under the Ministry of Health. Informed consent was obtained from the respondents at the time of data collection and they were assured of the confidentiality and anonymity of the information they provided. Each consented participant was made to sign an appropriate agreement form before the commencement of the interview.

## Results

**Figure 1.**
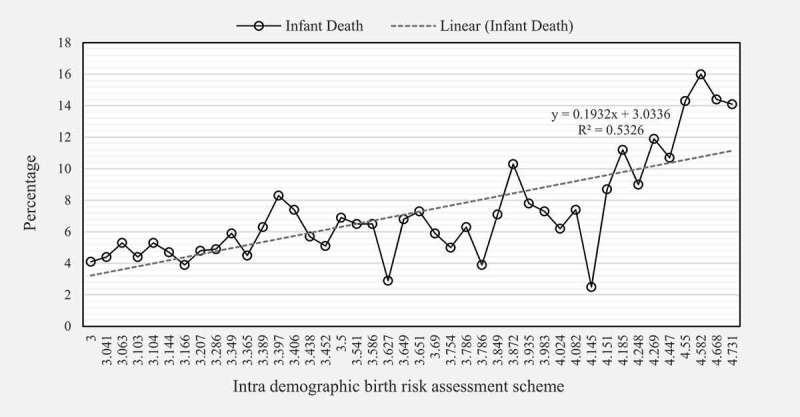
Percentage distribution of infant deaths by the intra-demographic birth risk assessment scheme.

In Figure 1, the data show that infant death increases as the IDBRAS increases. The fitted line indicates a positive relationship with infant mortality and that a unit increase in IDBRAS will increase the proportion of women who have experienced infant death in the 5 years before the survey by 19.32%. The range of IDBRAS was 3.000–4.731.

Socioeconomic factors such as region, education, place of residence, household wealth, religion, ethnicity and marital status were statistically significantly associated with IDBRAS (*p* < 0.001). About 24.6 and 4.2% of the women had medium- and high-risk births respectively. High IDBRAS was found most in the north-west (6.2%) and least in the south-west (1.3%) regions in Nigeria. Women with high IDBRAS were mostly found in the rural areas (5.0%), compared with 2.6% observed in the urban areas. The proportion of women with high IDBRAS falls consistently as the level of education and household wealth increases. It falls from 6.2% found among women with no formal education to merely 0.5% among women with higher education. The distribution of high IDBRAS was 5.9% among women with poor household wealth compared to 2.2% among women with the high household wealth. The prevalence of high IDBRAS was higher among the women who belong to the Islam (6.5%) religious denomination compared to 2.6% observed among the Christians. The proportion of women with high IDBRAS was greater among the Hausa/Fulani (6.0%) women than among either Igbo (2.8%) or Yoruba women (0.2%). High IDBRAS was more pronounced among currently married women (4.3%) than women who had never been in a union (0.2%) or who were formerly in a union (3.5%). However, medium IDBRAS was found most among women who had never married (51.2%). (Table 1).

**Table 1.** Percentage and mean distribution of intrademographic birth risk assessment scheme by socio-economic characteristics in Nigeria.

	IDBRAS			χ^2^-value
Socio-economic variables	Medium	High	Total women	(*p*-value)
Total	24.6	4.2	31,155	
Region				544.642*
North central	21.5	1.9	4555	(<0.001)
North east	27.9	5.5	6451	
North west	26.8	6.2	9823	
South east	25.9	3.3	2783	
South south	24.0	3.3	3715	
South west	17.1	1.3	3828	
Residence				187.579*
Urban	21.9	2.6	10,235	(<0.001)
Rural	26.0	5.0	20,920	
Education				480.261*
No education	26.0	6.2	14,624	(<0.001)
Primary	22.2	4.3	6367	
Secondary	25.9	1.5	8270	
Higher	17.3	0.5	1894	
Household wealth				354.187*
Poor	26.8	5.9	14,320	(<0.001)
Middle	25.2	3.8	6207	
Rich	21.4	2.2	10,628	
Religion				215.802*
Christianity	22.5	2.6	12,520	(<0.001)
Islam	26.1	5.3	18,169	
Others	19.8	6.5	308	
Ethnicity				518.975*
Hausa/Fulani	27.4	6.0	12,408	(<0.001)
Igbo	25.9	2.8	3374	
Yoruba	14.9	0.6	3397	
Others	24.2	3.8	11,976	
Marital status				240.361*
Never in union	51.2	0.2	592	(<0.001)
Currently in union	24.1	4.3	29,685	
Formerly in union	23.7	3.5	878	

*Significant at 0.1%; intra-demographic birth risk assessment scheme: IHRPAS.

As shown in the first model, the likelihood of infant mortality was 1.35 (95% CI = 1.22–1.48, *p* < 0.001) and 1.73 (95% CI = 1.48–2.02, *p* < 0.001) higher among women in medium and high IDBRAS categories respectively. This pattern was observed across the five models. The fifth model, which has the lowest −2log likelihood value (14,966.729), was the best model among the five models, where the chance of infant mortality was found to be 1.70 (95% CI = 1.33–2.16; *p* < 0.001) times higher among women with high IDBRAS than among their counterparts in the low IDBRAS group. The other predictors of infant mortality were place of residence, marital status, sex of the child and size of the child at birth. The hazard ratio of infant mortality was higher among rural women (HR = 1.30; 95% CI = 1.06–1.59, *p* < 0.001) than among urban women but significantly lower (HR = 0.60; 95% CI = 0.41–0.87, *p* < 0.001) among women currently in a union than among never-married women. Children being small in size (HR = 1.56, CI = 1.29–1.87) at the time delivery predisposed them to higher infant mortality than those who were bigger than the average size at the time delivery. Also, the infant death risk was 1.27 (HR = 1.27; 95% C.I = 1.11–1.46, *p* < 0.001) times higher among male children than female (Table 2).

**Table 2.** Models of the relationship between intra-demographic birth risk assessment scheme and infant mortality in Nigeria.

	aHR(95% CI)	aHR(95% CI)	aHR(95% CI)	aHR(95% CI)	aHR(95% CI)
Variables	Model 1	Model 2	Model 3	Model 4	Full model
Intra-demographic birth risk assessment scheme					
Low	1.00	1.00	1.00	1.00	1.00
Medium	1.35(1.22–1.48)*	1.33(1.21–1.46)*	1.11(0.95–1.30)	1.31(1.18–1.44)*	1.07(0.91–1.26)
High	1.73(1.48–2.02)*	1.69(1.44–1.97)*	1.73(1.36–2.19)*	1.67(1.42–1.96)*	1.70(1.33–2.16)*
Region					
North central	1.00	1.00			1.00
North east	1.03(0.88–1.20)	1.04(0.88–1.23)			1.14(0.87–1.49)
North west	1.12(0.97–1.28)	1.22(1.01–1.48)***			1.27(0.93–1.73)
South east	1.27(1.06–1.52)**	1.25(0.86–1.80)			1.34(0.76–2.32)
South south	0.94(0.78–1.13)	0.92(0.85–1.12)			0.98(0.72–1.33)
South west	0.97(0.81–1.17)	1.08(0.85–1.37)			1.19(0.80–1.75)
Residence					
Urban	1.00	1.00			1.00
Rural	1.20(1.08–1.32)*	1.22(1.08–1.39)**			1.30(1.06–1.59)**
Education					
No education	1.31(1.03–1.64)***	1.16(0.89–1.52)			0.93(0.61–1.39)
Primary	1.37(1.07–1.73)***	1.25(0.96–1.61)			1.11(0.75–1.64)
Secondary	1.21(0.95–1.53)	1.11(0.86–1.42)			1.02(0.70–1.47)
Higher	1.00	1.00			1.00
Household wealth					
Poor	1.21(1.09–1.34)*	1.04(0.89–1.21)			0.98(0.76–1.27)
Middle	1.09(0.95–1.24)	0.96(0.82–1.11)			0.97(0.75–1.23)
Rich	1.00	1.00			1.00
Religion					
Christianity	1.00				
Islam	0.93(0.85–1.02)				
Others	0.97(0.63–1.48)				
Ethnicity					
Hausa/Fulani	1.00	1.00			1.00
Igbo	1.15(1.01–1.33)***	1.29(0.89–1.85)			1.23(0.72–2.11)
Yoruba	0.89(0.75–1.04)	0.99(0.91–1.60)			0.85(0.54–1.33)
Others	0.98(0.88–1.08)	1.16(1.01–1.34)***			1.15(0.91–1.45)
Marital status					
NIU	1.00	1.00			1.00
CIU	0.62(0.47–0.83)**	0.66(0.48–0.89)**			0.60(0.41–0.87)**
FIU	0.81(0.56–1.15)	0.83(0.57–1.20)			0.70(0.43–1.13)
Cooking fuel					
Clean fuel	1.00				1.00
Biogas	1.15(1.01–1.31)***				1.02(0.78–1.33)
Sources of drinking water					
Improved	1.00				
Unimproved	1.09(0.99–1.18)				
Toilet facility					
Improved	1.00				1.00
Unimproved	1.14(1.05–1.25)**				1.02(0.87–1.19)
Tetanus injection during pregnancy					
No	1.00				
Yes	0.96(0.84–1.09)				
Number of ANC visits					
None	1.21(1.05–1.39)**		1.19(1.01–1.41)***		1.11(0.92–1.33)
1–3 visits	1.14(0.93–1.40)		1.16(0.94–1.44)		1.12(0.90–1.40)
≥4 visits	1.00		1.00		1.00
Place of delivery					
Home	1.00		1.00		1.00
Other	2.53(1.05–6.09)***		1.08(0.15–7.69)		0.92(0.12–6.54)
Health facility	0.97(0.88–1.07)		1.07(0.90–1.26)		1.06(0.87–1.29)
Prenatal attendant					
None	1.00				
Unskilled	1.39(0.83–2.34)				
Semi-skilled	1.00(0.79–1.27)				
Skilled	0.90(0.78–1.03)				
Delivery assistant					
None	1.00				
Unskilled	1.04(0.91–1.19)				
Semi-skilled	1.14(0.92–1.41)				
Skilled	0.99(0.86–1.14)				
Size at birth					
Small	1.67(1.49–1.88)*			1.64(1.45–1.85)*	1.56(1.29–1.87)*
Average	1.14(1.03–1.26)***			1.15(1.04–1.28)**	1.13(0.97–1.31)
> Average	1.00			1.00	1.00
Sex					
Male	1.17(1.07–1.27)*			1.18(1.08–1.29)*	1.27(1.11–1.46)*
Female	1.00			1.00	1.00
**−2log likelihood**		**39,384.266**	**15,621.562**	**36,602.092**	**14,966.729**

NIU, never in marital union; CIU, currently in marital union; NIU, never in marital union.

*Significant at 0.1%; **significant at 1.0%; ***significant at 5.0%.

**Figure 2.**
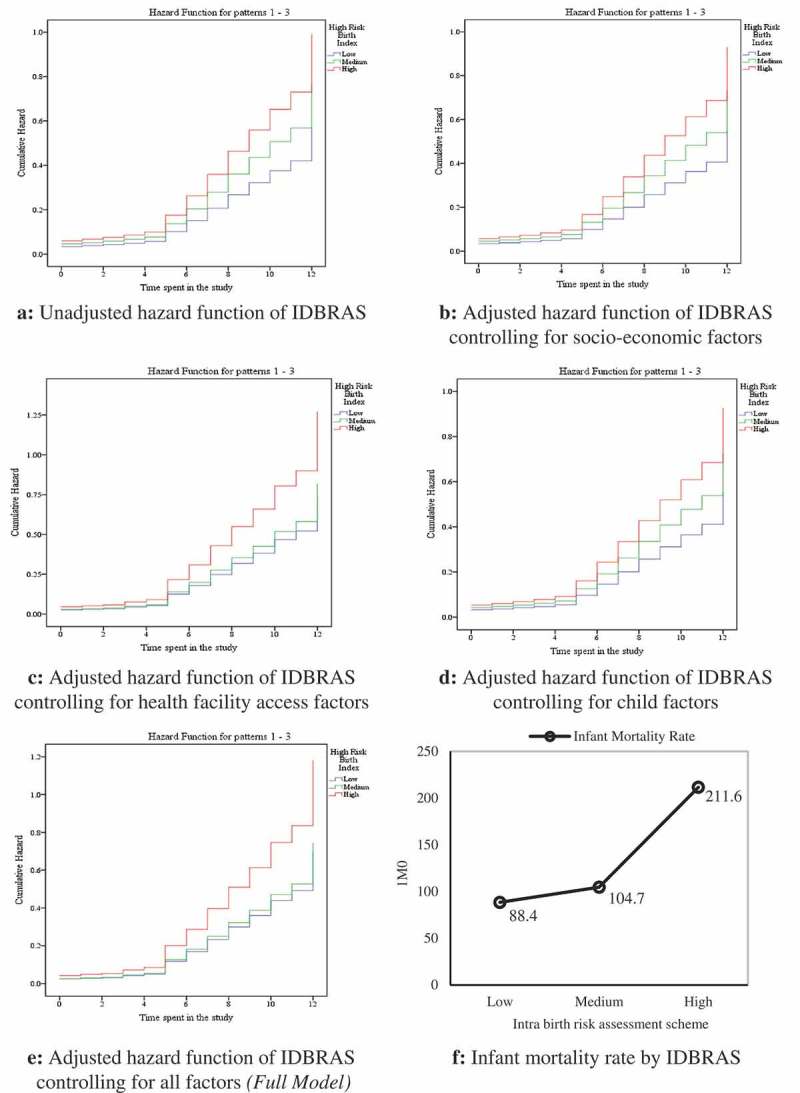
**(a)** Unadjusted hazard function of IDBRAS. (b) Adjusted hazard function of IDBRAS controlling for socio-economic factors. (c) Adjusted hazard function of IDBRAS controlling for health facility access factors. (d) Adjusted hazard function of IDBRAS controlling for child factors. (e) Adjusted hazard function of IDBRAS controlling for all factors (Full model). (f) Infant mortality rate by IDBRAS.

The data as shown in Figure 2(a–e) present the pattern of hazard function displayed by IDBRAS level when used in the five models fitted to examine its relationship with infant mortality. In the figures, the patterns displayed were similar with consistency in the arrangement of the infant mortality hazard function of IDBRAS across the models. High, medium and low IDBRAS was associated with higher infant mortality in that order. In Figure 2(f), the adjusted infant mortality rate was highest among children of women in high IDBRAS (211.6 per 1000 live births) compared with 104.7 per 1000 live births and 88.4 per 1000 live births found among women in medium and low IDBRAS respectively.

In Table 3, the unadjusted and adjusted probabilities of dying before the first birthday were displayed. The data showed that the refined probability of dying was lower than the unadjusted estimate across the IDBRAS categories. The adjusted probabilities of dying before age 1 were 0.083229, 0.097571 and 0.184299 among women with low, medium and high IDBRAS respectively. The parameter α in the Brass logit equation increases with increasing level of IDBRAS. Among women in low IDBRAS, the parameter was −0.20247, while it was −0.11509 and 0.253419 among women in medium and high respectively.

**Table 3.** Unadjusted and adjusted probability of dying (*q*(*x*)) before the first 12 months of life by intra-demographic birth risk assessment scheme.

Age (*x*)	Average parity/woman	Proportion of dead children	Multiplier ζ(*i)*	Probability of dying (*q*(*x*))	Logit of observed (*Y*(*x*))	Logit of standard (*Y*(*s*))	Adjusted (*Y*(*x*))	Adjusted *q*(*x*)
Low intra-demographic birth risk assessment scheme: α = −0.20247								
1	2.2529	0.1436	1.0266	**0.1474††**	−0.8775	−0.9972	−1.19963	**0.083229†**
2	2.3636	0.1209	1.0395	0.1257	−0.9699	−0.8053	−1.00779	0.117577
3	3.3393	0.1344	1.0014	0.1346	−0.9305	−0.7253	−0.92780	0.135217
5	4.6688	0.1426	1.0137	0.1445	−0.8891	−0.6514	−0.85385	0.153462
Medium intra-demographic birth risk assessment scheme: α = −0.11509								
1	1.3019	0.1473	1.0266	**0.1513††**	−0.8624	−0.9972	−1.11225	**0.097571†**
2	2.1381	0.1393	1.0395	0.1448	−0.8880	−0.8053	−0.92041	0.136955
3	4.0559	0.1675	1.0014	0.1677	−0.8010	−0.7253	−0.84042	0.156984
5	4.7825	0.1554	1.0137	0.1575	−0.8383	−0.6514	−0.76647	0.177563
High intra-demographic birth risk assessment scheme: α = 0.253419								
1	4.0000	0.3333	1.0266	**0.3422††**	−0.3268	−0.9972	−0.74374	**0.184299†**
2	4.4688	0.2657	1.0395	0.2762	−0.4816	−0.8053	−0.55190	0.249028
3	7.6119	0.2647	1.0014	0.2651	−0.5099	−0.7253	−0.47191	0.280129
5	8.2025	0.2932	1.0137	0.2972	−0.4303	−0.6514	−0.39796	0.310897

††, unadjusted probability of dying before age one; †, adjusted probability of dying before age one.

## Discussion

The persistent high level of infant and childhood mortality in Nigeria [9] requires urgent interventions, both medical and social, that can facilitate its quick reduction. Consequently, diverse quantitative and qualitative research has been conducted to investigate factors influencing mortality among children in Nigeria [14,15,17,19,29]. Despite the existing interesting findings from these studies and their impact on mortality and its associated causes through policies, a more scholarly approach is obligatory to enhance the survival chances of newborn babies. It was emphasized in Millennium Development Goals (MDGs) that childhood mortality should be reduced by 2015 [30]. Unfortunately, Nigeria is among the countries which failed to achieve this target at the deadline. The Sustainable Development Goals (SDGs) also support this agenda. Therefore, research on childhood mortality remains a discourse of global interest. The intra-demographic birth risk assessment scheme (IDBRAS) developed in this study is a new approach to measuring the risk associated with maternal age at childbirth, preceding birth interval and parity. The method combines these maternal demographic factors as a unit and examines its influence on infant mortality. In addition, the infant mortality rate was provided for the disaggregated IDBRAS.

The view that age of the mother at childbirth, birth interval and birth order are linked to infant mortality has been consistently reported in the literature [17,19–21]. In most of these studies, a higher risk of infant mortality was found to be associated with short and too long birth interval, births to women less than 19 years and above 35 years of age, first birth and parity four and higher births [17,19–21]. It is therefore expected that the combined effect of these factors should increase the risk of infant mortality. This is the situation found in the current study, where the hazard ratio of infant death was found to increase consistently as the level of IDBRAS increased. The refined estimates of the probability of dying before age one (_1_ *q*_0_) and infant mortality rate (IMR) obtained across the IDBRAS categories also followed this pattern. The _1_ *q*_0_ ranged between 0.0832 and 0.1843 while the range for IMR was between 88.4 per 1000 live births and 211.6 per 1000 live births. The lowest estimated IMR (88.4 per 1000 live births) was within the figure obtained in the Akinyemi and colleagues study, but higher than the national estimate for Nigeria (68 per 1000 live births) using the same data set [17]. In fact, the lowest IMR found in this study for women in the low IDBRAS category should have been lower than the national average reported in the Nigeria demographic health and survey report [1,9]. Different approach in methodologies and exclusion of women with missing information on the principal variables for IDBRAS computation could be responsible for this variation.

Since IDBRAS is a new method of assessing maternal health, examining the relationship between this index and socioeconomic factors will be invaluable to health programmers and policy-makers. In this study, region of residence, level of education, place of residence, household wealth, religion, ethnicity and marital status were identified socioeconomic factors associated with IDBRAS categorized as low, medium and high. Among mothers who had a delivery in the 5 years prior to the survey, those who are residents of the north-east part of Nigeria were most found to experience high IDBRAS. This is anticipated, since previous studies in Nigeria have found that women in this region mostly begin childbearing at younger ages and possibly continue childbearing throughout their childbearing period [9]. This is evident in their mean children-ever-born and total fertility rates often reported in the literature [9]. In such a situation, there is a feasibility of higher-parity women and high birth frequency resulting from shorter birth intervals. The inverse patterns in proportion of women with high IDBRAS exhibited by level of education and household wealth were similar and expected as found in previous studies [14,31]. More high IDBRAS was found among rural than among urban women and greater prevalence among married than unmarried women. These findings are in agreement with the earlier studies conducted in Nigeria and other parts of sub-Saharan Africa if IDBRAS was disentangled into its main components [5,31].

Apart from IDBRAS, which demonstrates a strong relationship with infant mortality after removing the effect of potential confounders, the other prognostic factors of infant mortality identified in this study were place of residence, marital status, sex of the child and size of the child at birth. Being a male child, residence in a rural area, small at delivery and a single mother predisposed children to higher childhood mortality in Nigeria. The direction of the relationship between these factors and infant mortality as established in this study is consistent with what is known in this area of research [5,16,31,32].

### Limitation

The cross-sectional nature of the data might not allow reporting of a causal relationship between IDBRAS and infant mortality in Nigeria. Therefore, researchers should interpret the findings from this study with caution.

## Conclusion

The risk and level of infant mortality increased as the level of IDBRAS increased in Nigeria. IDBRAS was an important predictor of infant mortality. Other predictors of infant mortality included place of residence, marital status, sex of the child and size of the child at birth. Maintaining a low level of IDBRAS will facilitate reduction in the infant mortality rate in Nigeria. The methodology involved in the formulation of IDBRAS is hereby recommended for use in disaggregating score-oriented variables into components. It is indeed understood that many factors, including maternal, environmental and biological, account for child survival. However, estimation of IDBRAS at a glance for a newborn baby can provide insight into the survival chances of the baby before 12 months of age. Therefore, in order to be more confident in the use of IDBRAS clear understanding of other confounders might be necessary.

## Paper context

The study used an innovative method to assess the level of infant mortality in Nigeria. The constructed ‘intra-demographic birth risk assessment scheme’ in this study was used for the first time in research history. Therefore, there is a need for future research to improve this new measure of a child’s survival risk.
